# (*S*)-*N*-Benzyl-2-methyl-1,2,3,4-tetra­hydro­isoquinoline-3-carboxamide

**DOI:** 10.1107/S1600536810050361

**Published:** 2010-12-11

**Authors:** Tricia Naicker, Thavendran Govender, Hendrik. G. Kruger, Glenn. E.M. Maguire

**Affiliations:** a School of Pharmacy and Pharmacology, University of KwaZulu Natal, Durban 4000, South Africa; b School of Chemistry, University of KwaZulu Natal, Durban 4000, South Africa

## Abstract

The structure of the title compound, C_18_H_20_N_2_O, at 173 K has hexa­gonal (*P*6_1_) symmetry. The *N*-containing six-membered ring assumes a half-chair conformation. In the crystal, inter­molecular N—H⋯O hydrogen bonding *via* the amide groups cross-link the mol­ecules along the *a* axis. The absolute configuration was confirmed by 2D NMR studies.

## Related literature

The title compound is a precursor to chiral ligands involving a tetra­hydro­isoquinoline backbone. For the application of these ligands as catalysts, see: Chakka *et al.* (2009 ▶); Peters *et al.* (2010 ▶); Naicker *et al.* (2010*a* ▶). For related structures, see: Chakka *et al.* (2010 ▶). For a related structure with the same chiral centre and conformation of the six-membered ring, see: Naicker *et al.* (2010*b* ▶).
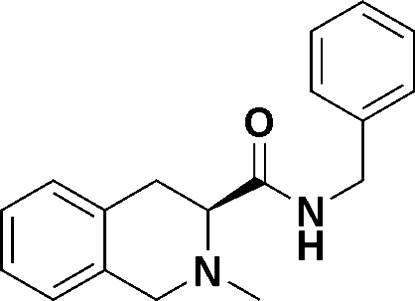


## Experimental

### 

#### Crystal data

C_18_H_20_N_2_O
*M* *_r_* = 280.36
Hexagonal, 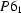

*a* = 10.1838 (13) Å
*c* = 25.965 (3) Å
*V* = 2332.1 (5) Å^3^
*Z* = 6
Mo *K*α radiation
μ = 0.08 mm^−1^
*T* = 173 K
0.22 × 0.12 × 0.03 mm

#### Data collection

Bruker Kappa DUO APEXII diffractometer
18777 measured reflections
1759 independent reflections
1358 reflections with *I* > 2σ(*I*)
*R*_int_ = 0.059

#### Refinement

*R*[*F* ^2^ > 2σ(*F* ^2^)] = 0.036
*wR*(*F* ^2^) = 0.088
*S* = 1.05
1759 reflections
195 parameters
2 restraints
H atoms treated by a mixture of independent and constrained refinement
Δρ_max_ = 0.14 e Å^−3^
Δρ_min_ = −0.14 e Å^−3^

### 

Data collection: *APEX2* (Bruker, 2006 ▶); cell refinement: *SAINT* (Bruker, 2006 ▶); data reduction: *SAINT*; program(s) used to solve structure: *SHELXS97* (Sheldrick, 2008 ▶); program(s) used to refine structure: *SHELXL97* (Sheldrick, 2008 ▶); molecular graphics: *OLEX2* (Dolomanov *et al.*, 2009 ▶); software used to prepare material for publication: *SHELXL97*.

## Supplementary Material

Crystal structure: contains datablocks I, global. DOI: 10.1107/S1600536810050361/hg2752sup1.cif

Structure factors: contains datablocks I. DOI: 10.1107/S1600536810050361/hg2752Isup2.hkl

Additional supplementary materials:  crystallographic information; 3D view; checkCIF report

## Figures and Tables

**Table 1:** Hydrogen-bond geometry (Å, °)

*D*—H⋯*A*	*D*—H	H⋯*A*	*D*⋯*A*	*D*—H⋯*A*
N2—H2⋯O1^i^	0.96 (2)	1.92 (2)	2.852 (3)	165 (3)

Symmetry code: (i) 
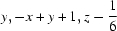
.

**Fig. 1.**
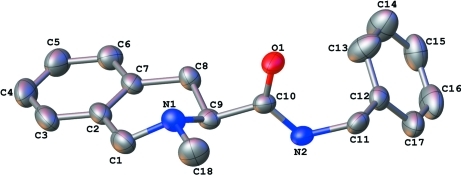
The molecular structure of compound 1 with the atom numbering scheme. Displacement ellipsoids are drawn at the 50% probability level. Hydrogen atoms have been omitted for clarity.

**Fig. 2.**
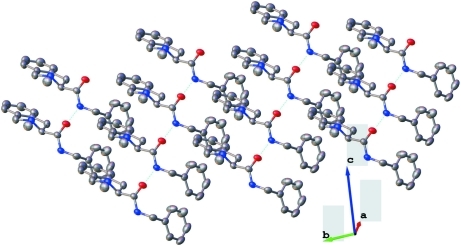
Hydrogen bonding interactions between atoms N2—H2···O1.

## supplementary crystallographic information

### Comment

The title compound (Fig. 1) is a precursor in the synthesis of novel chiral ligands involving a tetrahydroisoquinoline backbone. Recently, we have reported the application of these ligands as useful catalysts for transfer hydrogenation of prochiral ketones (Chakka *et al.*, 2009), Henry reactions, hydrogenation of olefins (Peters *et al.* 2010) and Diels-Alder reactions (Naicker *et al.*, 2010*a*).

Compound 1 was derived from commercially available *S-*phenyl glycine and formaldehyde. The absolute stereochemistry was confirmed to be S at the C9 position from proton NMR spectroscopy. (Peters *et al.* 2010).

From the crystal structure it is evident that the *N*-containing six membered ring assumes a half chair conformation (Fig. 1), in which the 1—N1—C9—C8 bond has a torsion angle of 68.7 (3)°. This observation is similar to analogous structures that we have reported recently (Chakka *et al.*, 2010) and (Naicker *et al.*, 2010*b*).

The molecule exhibits intermolecular hydrogen bonding, which involves the atom O1 which links the molecules together see Table 1 and Fig. 2.

### Experimental

(*S*)-2-methyl-1,2,3,4-tetrahydroisoquinoline-3-carboxylic acid (1.5 g, 7.8 mmol) was dissolved in DMF (15 ml) followed by addition of 1-ethyl-3-(3-dimethylaminopropyl) carbodiimide (EDC) hydrochloride (8.8 mmol), hydroxybenzotriazole (0.81 g, 8.3 mmol), a catalytic amount of 4-dimethylaminopyridine and benzyl amine (8.3 mmol). The reaction mixture was then stirred at room temperature until no more starting material could be detected by TLC analysis (approximately 1 h). The reaction mixture was poured into 30 volumes of chilled water; the mixture was then extracted twice with ethyl acetate. The extracts were combined, washed with 5% HCl (aq) to remove latent EDC urea, dried over anhydrous magnesium sulfate and then concentrated to dryness affording the crude product which was purified by column chromatography.

Melting point 91–95 *^o^*C. [*α*]^20^_D_ -7.93 (*c* 0.21 in CHCl_3_).

IR (neat) n_max_: 3281, 2923, 1646, 1548, 1454, 1240, 739, 696 cm^-1^.

^1^H NMR (400 MHz, CDCl_3_) δ = 2.78 (d, 3H), 3.12 (m, 2), 3.52 (t, 1H), 3.66 (m, 3H), 3.78 (d, 1H), 6.99 (d, 1H), 7.19 (m, 3H), 7.30 (m, 6H)

Recrystallization from EtOAc afforded colourless crystals suitable for X-ray analysis.

### Refinement

All hydrogen atoms on carbons were positioned geometrically with C—H distances ranging from 0.95 Å to 1.00 Å and refined as riding on their parent atoms, with *U*_iso_ (H) = 1.2 - 1.5 *U*_eq_ (C). The position of amine hydrogen H2 was located in the difference electron density maps and refined with simple bond length constraints. The Flack *x* parameter is -0.5 (15) without merging Friedel pairs, so Friedel pairs were merged at the final refinement.

### Crystal data

**Table:**

C_18_H_20_N_2_O	*D*_x_ = 1.198 Mg m^−^^3^
*M**_r_* = 280.36	Melting point: 365 K
Hexagonal, *P*6_1_	Mo *K*α radiation, λ = 0.71073 Å
Hall symbol: P 61	Cell parameters from 18777 reflections
*a* = 10.1838 (13) Å	θ = 2.3–27.2°
*c* = 25.965 (3) Å	µ = 0.08 mm^−^^1^
*V* = 2332.1 (5) Å^3^	*T* = 173 K
*Z* = 6	Needle, colourless
*F*(000) = 900	0.22 × 0.12 × 0.03 mm

### Data collection

**Table:**

Bruker Kappa DUO APEXII diffractometer	1358 reflections with *I* > 2σ(*I*)
Radiation source: fine-focus sealed tube	*R*_int_ = 0.059
graphite	θ_max_ = 27.2°, θ_min_ = 2.3°
0.5° φ scans and ω scans	*h* = −13→12
18777 measured reflections	*k* = −12→13
1759 independent reflections	*l* = −33→33

### Refinement

**Table:**

Refinement on *F*^2^	Secondary atom site location: difference Fourier map
Least-squares matrix: full	Hydrogen site location: inferred from neighbouring sites
*R*[*F*^2^ > 2σ(*F*^2^)] = 0.036	H atoms treated by a mixture of independent and constrained refinement
*wR*(*F*^2^) = 0.088	*w* = 1/[σ^2^(*F*_o_^2^) + (0.0377*P*)^2^ + 0.3273*P*] where *P* = (*F*_o_^2^ + 2*F*_c_^2^)/3
*S* = 1.05	(Δ/σ)_max_ < 0.001
1759 reflections	Δρ_max_ = 0.14 e Å^−^^3^
195 parameters	Δρ_min_ = −0.14 e Å^−^^3^
2 restraints	Extinction correction: *SHELXL97* (Sheldrick, 2008), Fc^*^=kFc[1+0.001xFc^2^λ^3^/sin(2θ)]^-1/4^
Primary atom site location: structure-invariant direct methods	Extinction coefficient: 0.0033 (7)

### Special details

**Table:**

Experimental. Half sphere of data collected using *SAINT* strategy (Bruker, 2006). Crystal to detector distance = 40 mm; combination of φ and ω scans of 0.5°, 30 s per °, 2 iterations.
Geometry. All e.s.d.'s (except the e.s.d. in the dihedral angle between two l.s. planes) are estimated using the full covariance matrix. The cell e.s.d.'s are taken into account individually in the estimation of e.s.d.'s in distances, angles and torsion angles; correlations between e.s.d.'s in cell parameters are only used when they are defined by crystal symmetry. An approximate (isotropic) treatment of cell e.s.d.'s is used for estimating e.s.d.'s involving l.s. planes.
Refinement. Refinement of *F*^2^ against ALL reflections. The weighted *R*-factor *wR* and goodness of fit *S* are based on *F*^2^, conventional *R*-factors *R* are based on *F*, with *F* set to zero for negative *F*^2^. The threshold expression of *F*^2^ > σ(*F*^2^) is used only for calculating *R*-factors(gt) *etc*. and is not relevant to the choice of reflections for refinement. *R*-factors based on *F*^2^ are statistically about twice as large as those based on *F*, and *R*- factors based on ALL data will be even larger.

### Fractional atomic coordinates and isotropic or equivalent isotropic displacement parameters (Å^2^)

**Table:**

	*x*	*y*	*z*	*U*_iso_*/*U*_eq_	
O1	0.73695 (19)	0.77313 (19)	0.97396 (7)	0.0450 (5)	
N1	0.9118 (2)	1.1026 (2)	0.98992 (8)	0.0385 (5)	
N2	0.7085 (2)	0.8382 (2)	0.89310 (8)	0.0362 (5)	
H2	0.747 (3)	0.915 (2)	0.8671 (9)	0.051 (8)*	
C1	1.0391 (3)	1.2586 (3)	0.98885 (11)	0.0465 (7)	
H1A	1.0402	1.3099	1.0214	0.056*	
H1B	1.0241	1.3139	0.9602	0.056*	
C2	1.1898 (3)	1.2671 (3)	0.98225 (10)	0.0406 (6)	
C3	1.3244 (4)	1.4003 (3)	0.99539 (12)	0.0537 (8)	
H3	1.3197	1.4848	1.0087	0.064*	
C4	1.4632 (3)	1.4096 (3)	0.98919 (13)	0.0595 (8)	
H4	1.5535	1.4999	0.9984	0.071*	
C5	1.4714 (3)	1.2876 (4)	0.96964 (13)	0.0581 (8)	
H5	1.5671	1.2941	0.9652	0.070*	
C6	1.3402 (3)	1.1566 (3)	0.95657 (11)	0.0463 (7)	
H6	1.3461	1.0725	0.9436	0.056*	
C7	1.1987 (3)	1.1457 (3)	0.96216 (10)	0.0374 (6)	
C8	1.0561 (3)	1.0021 (3)	0.94725 (11)	0.0378 (6)	
H8A	1.0356	0.9225	0.9730	0.045*	
H8B	1.0718	0.9668	0.9135	0.045*	
C9	0.9188 (3)	1.0240 (3)	0.94379 (10)	0.0345 (5)	
H9	0.9283	1.0859	0.9126	0.041*	
C10	0.7785 (3)	0.8671 (3)	0.93857 (10)	0.0343 (5)	
C11	0.5866 (3)	0.6863 (3)	0.87851 (10)	0.0393 (6)	
H11A	0.5110	0.6958	0.8573	0.047*	
H11B	0.5349	0.6288	0.9100	0.047*	
C12	0.6459 (3)	0.6001 (3)	0.84871 (9)	0.0373 (6)	
C13	0.7331 (4)	0.5489 (4)	0.87268 (12)	0.0623 (9)	
H13	0.7528	0.5655	0.9085	0.075*	
C14	0.7920 (4)	0.4739 (4)	0.84516 (14)	0.0685 (10)	
H14	0.8523	0.4402	0.8622	0.082*	
C15	0.7643 (4)	0.4478 (4)	0.79358 (13)	0.0615 (9)	
H15	0.8053	0.3967	0.7747	0.074*	
C16	0.6769 (4)	0.4962 (4)	0.76946 (13)	0.0680 (10)	
H16	0.6565	0.4782	0.7337	0.082*	
C17	0.6180 (4)	0.5713 (3)	0.79703 (12)	0.0529 (7)	
H17	0.5568	0.6037	0.7798	0.063*	
C18	0.7705 (3)	1.1059 (4)	0.99296 (14)	0.0573 (8)	
H18A	0.6846	1.0019	0.9935	0.086*	
H18B	0.7619	1.1594	0.9629	0.086*	
H18C	0.7700	1.1586	1.0245	0.086*	

### Atomic displacement parameters (Å^2^)

**Table:**

	*U*^11^	*U*^22^	*U*^33^	*U*^12^	*U*^13^	*U*^23^
O1	0.0379 (9)	0.0402 (10)	0.0409 (10)	0.0076 (8)	−0.0062 (8)	0.0136 (8)
N1	0.0387 (12)	0.0377 (11)	0.0414 (12)	0.0208 (10)	0.0035 (9)	0.0012 (9)
N2	0.0370 (11)	0.0366 (11)	0.0349 (11)	0.0183 (9)	−0.0024 (9)	0.0071 (9)
C1	0.0571 (16)	0.0354 (14)	0.0488 (16)	0.0245 (13)	−0.0015 (13)	−0.0017 (12)
C2	0.0453 (14)	0.0287 (12)	0.0408 (14)	0.0133 (11)	−0.0010 (12)	0.0058 (11)
C3	0.065 (2)	0.0303 (14)	0.0522 (18)	0.0137 (14)	−0.0029 (14)	0.0044 (12)
C4	0.0422 (16)	0.0433 (16)	0.065 (2)	0.0006 (13)	−0.0056 (15)	0.0107 (15)
C5	0.0371 (15)	0.0565 (18)	0.067 (2)	0.0128 (14)	−0.0042 (14)	0.0108 (16)
C6	0.0365 (14)	0.0439 (15)	0.0532 (17)	0.0162 (12)	0.0031 (12)	0.0094 (13)
C7	0.0381 (13)	0.0290 (12)	0.0388 (13)	0.0121 (11)	0.0011 (11)	0.0073 (11)
C8	0.0336 (12)	0.0299 (12)	0.0459 (14)	0.0130 (11)	0.0036 (11)	−0.0001 (11)
C9	0.0349 (13)	0.0321 (12)	0.0340 (12)	0.0149 (10)	0.0029 (10)	0.0071 (10)
C10	0.0315 (12)	0.0360 (13)	0.0356 (13)	0.0170 (10)	0.0005 (10)	0.0067 (10)
C11	0.0295 (13)	0.0442 (14)	0.0403 (14)	0.0156 (11)	−0.0071 (11)	0.0036 (11)
C12	0.0300 (12)	0.0325 (13)	0.0375 (14)	0.0067 (10)	0.0004 (11)	0.0021 (11)
C13	0.073 (2)	0.097 (3)	0.0420 (17)	0.061 (2)	−0.0073 (15)	−0.0093 (16)
C14	0.069 (2)	0.089 (3)	0.067 (2)	0.054 (2)	−0.0099 (18)	−0.0217 (19)
C15	0.0525 (18)	0.0532 (18)	0.064 (2)	0.0151 (15)	0.0066 (16)	−0.0209 (16)
C16	0.085 (3)	0.0545 (19)	0.0404 (16)	0.0168 (18)	−0.0052 (17)	−0.0131 (15)
C17	0.0631 (19)	0.0404 (15)	0.0440 (16)	0.0174 (14)	−0.0140 (14)	−0.0050 (13)
C18	0.0526 (17)	0.067 (2)	0.065 (2)	0.0395 (16)	0.0052 (15)	−0.0012 (16)

### Geometric parameters (Å, °)

**Table:**

O1—C10	1.239 (3)	C8—H8A	0.9900
N1—C18	1.458 (3)	C8—H8B	0.9900
N1—C9	1.462 (3)	C9—C10	1.527 (3)
N1—C1	1.465 (3)	C9—H9	1.0000
N2—C10	1.334 (3)	C11—C12	1.504 (4)
N2—C11	1.469 (3)	C11—H11A	0.9900
N2—H2	0.957 (10)	C11—H11B	0.9900
C1—C2	1.503 (4)	C12—C17	1.373 (4)
C1—H1A	0.9900	C12—C13	1.383 (4)
C1—H1B	0.9900	C13—C14	1.383 (4)
C2—C7	1.386 (4)	C13—H13	0.9500
C2—C3	1.406 (4)	C14—C15	1.367 (5)
C3—C4	1.378 (4)	C14—H14	0.9500
C3—H3	0.9500	C15—C16	1.365 (5)
C4—C5	1.382 (5)	C15—H15	0.9500
C4—H4	0.9500	C16—C17	1.385 (5)
C5—C6	1.377 (4)	C16—H16	0.9500
C5—H5	0.9500	C17—H17	0.9500
C6—C7	1.396 (4)	C18—H18A	0.9800
C6—H6	0.9500	C18—H18B	0.9800
C7—C8	1.508 (4)	C18—H18C	0.9800
C8—C9	1.524 (3)		
			
C18—N1—C9	112.1 (2)	C8—C9—C10	107.39 (19)
C18—N1—C1	109.0 (2)	N1—C9—H9	109.5
C9—N1—C1	108.61 (19)	C8—C9—H9	109.5
C10—N2—C11	122.5 (2)	C10—C9—H9	109.5
C10—N2—H2	119.4 (18)	O1—C10—N2	123.2 (2)
C11—N2—H2	117.6 (18)	O1—C10—C9	121.5 (2)
N1—C1—C2	112.9 (2)	N2—C10—C9	115.3 (2)
N1—C1—H1A	109.0	N2—C11—C12	111.9 (2)
C2—C1—H1A	109.0	N2—C11—H11A	109.2
N1—C1—H1B	109.0	C12—C11—H11A	109.2
C2—C1—H1B	109.0	N2—C11—H11B	109.2
H1A—C1—H1B	107.8	C12—C11—H11B	109.2
C7—C2—C3	119.0 (3)	H11A—C11—H11B	107.9
C7—C2—C1	120.8 (2)	C17—C12—C13	117.6 (3)
C3—C2—C1	120.2 (3)	C17—C12—C11	121.8 (3)
C4—C3—C2	120.6 (3)	C13—C12—C11	120.6 (2)
C4—C3—H3	119.7	C12—C13—C14	120.9 (3)
C2—C3—H3	119.7	C12—C13—H13	119.5
C3—C4—C5	120.1 (3)	C14—C13—H13	119.5
C3—C4—H4	119.9	C15—C14—C13	120.6 (3)
C5—C4—H4	119.9	C15—C14—H14	119.7
C6—C5—C4	119.7 (3)	C13—C14—H14	119.7
C6—C5—H5	120.2	C16—C15—C14	119.2 (3)
C4—C5—H5	120.2	C16—C15—H15	120.4
C5—C6—C7	121.0 (3)	C14—C15—H15	120.4
C5—C6—H6	119.5	C15—C16—C17	120.2 (3)
C7—C6—H6	119.5	C15—C16—H16	119.9
C2—C7—C6	119.6 (2)	C17—C16—H16	119.9
C2—C7—C8	120.0 (2)	C12—C17—C16	121.5 (3)
C6—C7—C8	120.4 (2)	C12—C17—H17	119.3
C7—C8—C9	112.5 (2)	C16—C17—H17	119.3
C7—C8—H8A	109.1	N1—C18—H18A	109.5
C9—C8—H8A	109.1	N1—C18—H18B	109.5
C7—C8—H8B	109.1	H18A—C18—H18B	109.5
C9—C8—H8B	109.1	N1—C18—H18C	109.5
H8A—C8—H8B	107.8	H18A—C18—H18C	109.5
N1—C9—C8	109.34 (19)	H18B—C18—H18C	109.5
N1—C9—C10	111.7 (2)		
			
C18—N1—C1—C2	−176.3 (2)	C1—N1—C9—C10	−172.63 (19)
C9—N1—C1—C2	−53.9 (3)	C7—C8—C9—N1	−48.2 (3)
N1—C1—C2—C7	20.3 (4)	C7—C8—C9—C10	−169.6 (2)
N1—C1—C2—C3	−161.2 (2)	C11—N2—C10—O1	−7.5 (4)
C7—C2—C3—C4	−1.0 (4)	C11—N2—C10—C9	170.4 (2)
C1—C2—C3—C4	−179.6 (3)	N1—C9—C10—O1	−53.7 (3)
C2—C3—C4—C5	0.4 (5)	C8—C9—C10—O1	66.2 (3)
C3—C4—C5—C6	−0.2 (5)	N1—C9—C10—N2	128.3 (2)
C4—C5—C6—C7	0.8 (4)	C8—C9—C10—N2	−111.8 (2)
C3—C2—C7—C6	1.6 (4)	C10—N2—C11—C12	−94.2 (3)
C1—C2—C7—C6	−179.9 (2)	N2—C11—C12—C17	−109.0 (3)
C3—C2—C7—C8	−179.4 (2)	N2—C11—C12—C13	70.0 (3)
C1—C2—C7—C8	−0.8 (4)	C17—C12—C13—C14	1.2 (5)
C5—C6—C7—C2	−1.5 (4)	C11—C12—C13—C14	−177.9 (3)
C5—C6—C7—C8	179.4 (3)	C12—C13—C14—C15	−0.4 (6)
C2—C7—C8—C9	14.7 (3)	C13—C14—C15—C16	−0.3 (5)
C6—C7—C8—C9	−166.2 (2)	C14—C15—C16—C17	0.3 (5)
C18—N1—C9—C8	−170.9 (2)	C13—C12—C17—C16	−1.1 (4)
C1—N1—C9—C8	68.7 (2)	C11—C12—C17—C16	177.9 (3)
C18—N1—C9—C10	−52.2 (3)	C15—C16—C17—C12	0.4 (5)

### Hydrogen-bond geometry (Å, °)

**Table:**

*D*—H···*A*	*D*—H	H···*A*	*D*···*A*	*D*—H···*A*
N2—H2···O1^i^	0.96 (2)	1.92 (2)	2.852 (3)	165 (3)

Symmetry codes: (i) *y*, −*x*+*y*+1, *z*−1/6.
